# Genome-Wide Identification and Comparative Analysis of *ARF* Family Genes in Three Apiaceae Species

**DOI:** 10.3389/fgene.2020.590535

**Published:** 2021-01-13

**Authors:** Qiaoying Pei, Nan Li, Qihang Yang, Tong Wu, Shuyan Feng, Xuehuan Feng, Zange Jing, Rong Zhou, Ke Gong, Tong Yu, Zhen Wang, Xiaoming Song

**Affiliations:** ^1^College of Life Sciences/Center for Genomics and Bio-computing, North China University of Science and Technology, Tangshan, China; ^2^Food Science and Technology Department, University of Nebraska–Lincoln, Lincoln, NE, United States; ^3^College of Agriculture and Life Science, Kunming University, Kunming, China; ^4^Department of Food Science, Aarhus University, Aarhus, Denmark; ^5^Suzhou Polytechnic Institute of Agriculture, Suzhou, China; ^6^School of Life Science and Technology, Center for Informational Biology, University of Electronic Science and Technology of China, Chengdu, China

**Keywords:** *ARF* gene family, orthologous and paralogous genes, gene duplication and loss, expression pattern, Apiaceae species, phylogenetic analysis

## Abstract

The family Apiaceae includes many important vegetables and medicinal plants. Auxin response factors (ARFs) play critical roles in regulating plant growth and development. Here, we performed a comprehensive analysis of the *ARF* gene family in three Apiaceae species, celery, coriander, and carrot, and compared the results with the *ARF* gene family of lettuce, Arabidopsis, and grape. We identified 156 *ARF* genes in all six species and 89 genes in the three Apiaceae species, including 28, 34, and 27 in celery, coriander, and carrot, respectively. The paralogous gene number in coriander was far greater than that in carrot and celery. Our analysis revealed that *ARF* genes of the three Apiaceae species in 34 branches of the phylogenetic tree underwent significant positive selection. Additionally, our findings indicated that whole-genome duplication played an important role in *ARF* gene family expansion. Coriander contained a greater number of *ARF* genes than celery and carrot because of more gene duplications and less gene losses. We also analyzed the expression of *ARF* genes in three tissues by RNA-seq and verified the results by quantitative real-time PCR. Furthermore, we found that several paralogous genes exhibited divergent expression patterns. Overall, this study provides a valuable resource for exploring how *ARF* family genes regulate plant growth and development in other plants. Since this is the first report of the *ARF* gene family in Apiaceae, our results will serve as a guide for comparative and functional analyses of *ARF* and other gene families in Apiaceae.

## Introduction

Celery (*Apium graveolens*), coriander (*Coriandrum sativum*), and carrot (*Daucus carota*) are three typical members of Apiaceae family. They grow all over the world and are especially famous for their fragrance and medicinal value. Celery is a multipurpose plant, which used as a vegetable and medicinal herb for treating diseases (Ballmer-Weber et al., 2002; Maljaei et al., 2019). Coriander contains many bioactive phytochemicals and has also been used as a traditional medicine (Prachayasittikul et al., 2018). Carrot is one of the most important root vegetables around the world and is valued for its high content of beta-carotene, an essential component of vitamin A (Ahmad et al., 2019). Above all, these three Apiaceae crops are of high economical value, given their medicinal and edible properties, and therefore are a major source of income for growers.

The phytohormone auxin plays a very significant role in regulating not only plant developmental processes, such as apical dominance, later root initiation, and vascular differentiation, but also in cellular processes, including cell division, expansion, and differentiation (Wang et al., 2007; Xing et al., 2011). Auxin Response Factors (ARFs) regulate the expression of auxin-responsive genes during plant growth. The auxin response signal is transmitted to related response genes via auxin response elements (AuxREs) (Wan et al., 2014).

*ARF1* was the first *ARF* gene identified in *Arabidopsis thaliana* using a yeast one-hybrid screen (Ulmasov et al., 1997). A total of 23 *ARF* genes have been identified in Arabidopsis to date (Okushima et al., 2005). With the sequencing of genomes, more *ARF* family genes have been detected in plants. For example, 39 *ARF* genes were identified in poplar (*Populus trichocarpa*) (Kalluri et al., 2007), 25 in rice (*Oryza sativa*) (Wang et al., 2007), 19 in grape (*Vitis vinifera*) (Wan et al., 2014), 19 in sweet orange (*Citrus* × *sinensis*) (Li et al., 2015), 31 in Chinese cabbage (*Brassica rapa* subsp. *pekinensis*) (Mun et al., 2012), 20 in pineapple (*Ananas comosus*) (Su et al., 2017), 20 in barley (*Hordeum vulgare*) (Tombuloglu, 2019), 31 in maize (*Zea mays*) (Xing et al., 2011), 17 in tomato (*Solanum lycopersicum*) (Kumar et al., 2011), 31 in apple (*Malus domestica*) (Luo et al., 2014), 17 in physic nut (*Jatropha curcas*) (Tang et al., 2018), and 19 in pepper (*Capsicum annuum*) (Zhang et al., 2017). However, a comprehensive analysis of *ARF* gene family in Apiaceae species has not yet been reported. Recently, more sequencing data of Apiaceae have been released, thus serving as a valuable resource for further analysis of the *ARF* gene family (Iorizzo et al., 2016; Song et al., 2020b).

In this study, we performed a comprehensive and systematic analysis of the *ARF* gene family in three representative species of Apiaceae (celery, coriander, and carrot), with the aim to (i) identify *ARF* gene family members; (ii) classify these members based on phylogenetic relationship; (iii) map *ARF* genes to chromosomes; (iv) identify the paralogous and orthologous genes; (v) explore *ARF* gene loss and duplication; and (vi) explore the gene expression patterns in three tissues of celery and coriander.

## Materials and Methods

### Genome Sequence Retrieval and ARF Gene Identification

Whole genome sequences of coriander and celery were retrieved from the Coriander Genome Database (CGDB^1^) (Song et al., 2020a). The genome sequence of Arabidopsis was downloaded from the TAIR database^2^. Genome sequences of carrot (v2.0), lettuce (v5.0), and grape (Genoscope.12X) were downloaded from Phytozome (Jaillon et al., 2007; Iorizzo et al., 2016; Reyes-Chin-Wo et al., 2017).

The Pfam database was used to identify *ARF* family genes using the identifier PF06507 with an *e*-value < 1e-4 (Punta et al., 2012). Furthermore, the conserved domain database (CDD) and simple modular architecture research tool (SMART) were used to verify the identified genes (Marchler-Bauer et al., 2009; Letunic et al., 2012).

### Phylogenetic Analysis of ARFs

Amino acid sequences of ARFs of celery, coriander, carrot, Arabidopsis, lettuce, and grape were used for phylogenetic analysis. First, sequences were aligned using ClustalW (Li, 2003). Then, the multiple sequence alignment was restored in the PHYLIP format. Finally, a phylogenetic tree was constructed using the maximum likelihood (ML) method with IQ-TREE (Nguyen et al., 2015), based on JTT + F + R8 model, with 1,000 bootstrap replications.

### Chromosomal Location, Gene Structure, and Conserved Motif Analysis of ARF Genes

The chromosomal location of each *ARF* gene was retrieved from general feature format (gff) file, and the chromosome number, start position and end position of each gene were extracted using a Perl script. The chromosome information file was submitted to the MapChart to display the distribution of each gene (Voorrips, 2002).

The structure of *ARF* genes was drawn using Gene Structure Displayer Server 2.0 (GSDS) (Hu et al., 2015). The gff file of *ARF* gene was submitted to GSDS to illustrate the positions of exons, introns, and untranslated regions (UTRs). Conserved motif analysis was performed using the Multiple Expression motifs (Em) for Motif Elicitation (MEME) (Bailey et al., 2009).

### Analysis of ARF Gene Duplication and Loss

Orthologous and paralogous genes were identified using the OrthoMCL, with an *e*-value of 1e-5 (Li et al., 2003). The relationship among *ARF* gene orthologs and paralogs was illustrated using the Circos (Krzywinski et al., 2009). Gene duplication and gene loss analyses were performed using the Notung2.9 (Stolzer et al., 2012).

### Analysis of Collinearity and Duplication Type

The MCScanX was used to conduct collinearity analysis (Wang et al., 2012). Amino acid sequences were analyzed using the Blastp, with an *e*-value set at 1.0 × 10^–5^. Then, collinear blocks were detected by submitting the whole genome gff and Blastp result to the MCScanX. The duplicate_gene_classifier sub-program was used to identify the duplication type.

### Evolutionary Analysis of ARF Genes

Coding sequences (CDSs) of orthologous *ARF* gene pairs were aligned using the ClustalW, and the alignment file was transformed into the axt format file. The synonymous (Ks) and non-synonymous (Ka) substitution rates were calculated using the Ka/Ks_calculator 2.0 (Wang et al., 2010). The divergence time (T) was estimated using the equation, *T* = Ks/2r. The “r” represents neutral substitution rate (5.2 × 10^–9^ substitutions per site per year) (Song et al., 2020b).

### Selective Pressure Analysis of ARF Gene Family

The selective pressure analysis was performed using the PAML4.9 (Yang, 2007). The ML method and codon substitution models were adopted to test the likelihood rate of positive selection. Firstly, CDSs of *ARF* genes were aligned using the ClustalW. Then, each branch of the phylogenetic tree constructed by the PhyML3.0 using amino acid sequences was analyzed to speculate ω (the ratio of non-synonymous to synonymous distances) (Guindon et al., 2010). The M0, M1, M7, and M8 models were used to calculate variation sites.

### ARF Gene Expression Analysis Using RNA-Seq

RNA-seq data of *ARF* gene expression in three tissues (root, petiole, and leaf) of celery and coriander (each with three replicates) were obtained from our previous study (Song et al., 2020a). The RNA-seq data were deposited in the Genome Sequence Archive (GSA) of the BIG Data Center^3^ under the accession numbers CRA001996 and CRA001658. The expression data expressed as Fragments Per Kilobase of transcript sequence per Millions base pairs (FPKM) were log2-transformed for cluster analysis, as described previously (Song et al., 2014b). Hierarchical clustering analysis was conducted using the TBtools (Chen et al., 2020).

### Verification of RNA-Seq Data

The RNA-seq data of celery and coriander *ARF* genes were verified by quantitative real-time PCR (qRT-PCR). Total RNA was extracted from each sample using the RNA Kit (Tiangen, Beijing, China), and the mRNA was transcribed into cDNA using the PrimeScript cDNA Synthesis Kit (TaKaRa, Dalian, China). The resulting cDNA was used as a template for qRT-PCR, which was performed on the CFX96^TM^ Real-Time System (Bio-Rad, Beijing, China) using sequence-specific primers (Supplementary Table 1), with three replicates for each gene as described previously (Song et al., 2014a, 2016).

## Results

### Identification, Phylogenetic Analysis, and Classification of ARF Genes

We identified 28, 34, and 27 *ARF* genes in celery, coriander, and carrot, respectively, and renamed these genes according to their order on chromosomes (Supplementary Tables 2, 3). Lettuce shows the closest relationship with Apiaceae, and its genome sequence has been released (Reyes-Chin-Wo et al., 2017). Additionally, the *ARF* gene family of Arabidopsis and grape has been analyzed in detail (Okushima et al., 2005; Wei et al., 2006; Wan et al., 2014). Comparative analysis of the *ARF* genes of celery, coriander, and carrot with the Arabidopsis, lettuce, and grape genomes revealed 22, 26, and 19 *ARF* genes in the latter three species, respectively.

**FIGURE 1:**
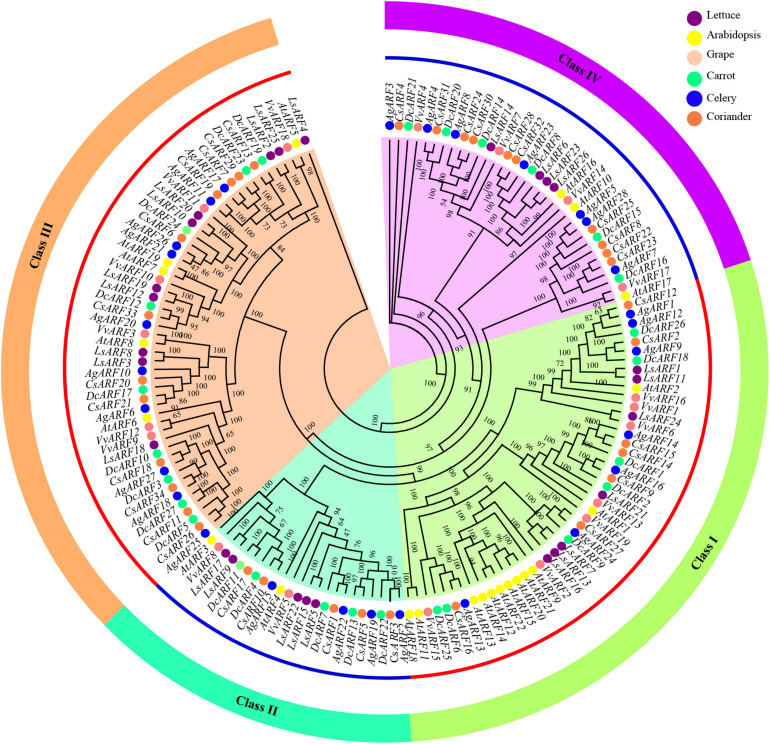
Phylogenetic analysis of ARF amino acid sequences in three Apiaceae species (carrot, celery, and coriander), lettuce, grape, and Arabidopsis. The phylogenetic tree was generated using the IQ-TREE software with the maximum likelihood (ML) based on the JTT + F + R8 model and 1,000 bootstrap replications.

To explore the evolutionary history and relationship of the *ARF* gene family, we constructed a phylogenetic tree using 156 ARF amino acid sequences from six species, including Arabidopsis, lettuce, grape, celery, coriander, and carrot (Figure 1). According to the phylogenetic analysis, all ARFs were divided into four classes (I − IV), based on the topology and classification in grape. Classes I and III contained a greater number of ARFs than classes II and IV. Interestingly, the number of Arabidopsis ARFs in one branch of class I was notably higher than that of other species, and eight Arabidopsis ARFs (AtARF9, AtARF12–15, AtARF20–22) clustered together.

We further constructed another phylogenetic tree using ARF sequences of celery, coriander, and carrot (Supplementary Figure 1). ARFs were also divided into four classes. The classification of ARFs among three Apiaceae species was highly consistent with that of six plant species (Figure 1). Additionally, classification within the Apiaceae showed that class III contained the highest number of ARFs (31), followed by class IV (23), class I (21), and class II (14).

### ARF Gene Structure Analysis and Conserved Motif Identification

To further explore the conservation of *ARF* family genes, we analyzed the gene structure and motifs. Full-length cDNA sequences of *ARF* genes were compared with the corresponding genomic sequences using the GSDS program (Supplementary Figure 2A). The number of exons in *ARF* genes varied from 1 − 15. In class I, most *ARF* genes contained 10–14 exons, with the exception of *AgARF1* and *CsARF16*. In class III, most *ARF* genes harbored 10–15 exons, except *AgARF26* and *CsARF29*. In class IV, *ARF* genes contained only 3–4 exons, which was far less than the number of exons in other classes. We found that the position and number of exons and introns in genes belonging to the same class or subclass were similar. This finding supports the phylogenetic relationship of *ARF* family genes.

We also analyzed conserved motifs in ARF genes using MEME. Eight motifs were detected (Supplementary Figure 2B), of which four (motifs 1, 2, 3, and 6) were common to almost all *ARF* genes. In classes I–III, most *ARF* genes contained motifs 1–4 and motifs 6–8. Interestingly, *AgARF1* contained only motif 4. In class IV, all *ARF* genes carried motifs 1–4 and motifs 6–8, except *CsARF30*. However, motif 5 was absent from almost all genes in class IV. In conclusion, motif 5 was lost in most *ARF* genes of celery, carrot, and coriander, while motifs 1–4 and motifs 6–8 were highly conserved in three Apiaceae species.

### Chromosomal Distribution of ARF Family Genes

In celery, 25 out of 28 *ARF* genes were unevenly distributed on 10 chromosomes, and three genes could not be mapped to any chromosome (Supplementary Figure 3A). Chromosomes 3 and 11 harbored the highest number of *ARF* genes (four genes), but no gene was detected on chromosome 8. Several genes, such as *AgARF4*, *AgARF8*, *AgARF9*, *AgARF12*, and *AgARF25*, were located at the end of the chromosomes 2, 3, 4, 5, and 11, respectively.

In coriander, 28 out of 34 *ARF* genes were unevenly distributed on eight chromosomes, while six *ARF* genes could not be mapped to any chromosome (Supplementary Figure 3B). Chromosome 10 contained the highest number of *ARF* genes (nine genes), but no *ARF* gene was found on chromosomes 6 and 8. Two *ARF* gene clusters were detected at the ends of chromosome 10, which might be caused by gene duplication. *CsARF3*, *CsARF14*, and *CsARF20* were located very close to *CsARF4*, *CsARF15*, and *CsARF21*, respectively.

In carrot, all 27 *ARF* genes were mapped to one of the eight chromosomes (Supplementary Figure 3C). Chromosome 2 carried the highest number of *ARF* genes (seven genes), followed by chromosomes 4 and 5, whereas chromosomes 3 and 8 harbored only one *ARF* gene each.

### Identification of Orthologous and Paralogous Gene Pairs

**FIGURE 2:**
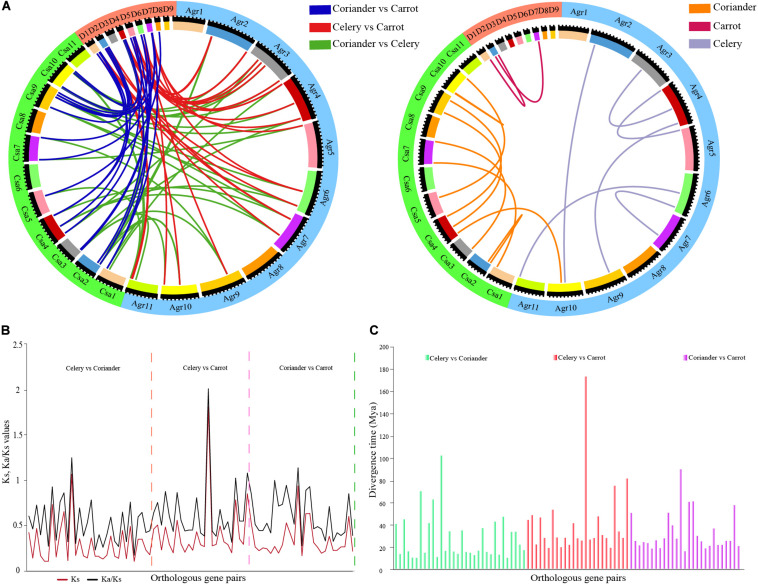
Analysis of paralogous and orthologous *ARF* gene pairs. **(A)** Circos plot of *ARF* gene paralogs and orthologs in three Apiaceae species. **(B)** Ks and Ka/Ks values of orthologous *ARF* gene pairs between any two of three Apiaceae species. **(C)** Divergence time of orthologous *ARF* gene pairs between any two of three Apiaceae species.

Forty orthologous gene pairs were detected between celery and coriander, while eight gene pairs could not be mapped to any chromosome. Twenty-six orthologous genes were found between celery and carrot, of which one pair could not be mapped. Totally, 29 orthologous gene pairs were identified between coriander and carrot (Figure 2A and Supplementary Table 4). Next, we identified paralogous gene pairs to explore the relationship of *ARF* genes within species (Supplementary Table 5). A total of eight and 12 paralogous gene pairs were detected in celery and coriander, respectively; however, only two paralogous gene pairs were identified in carrot.

We calculated the Ka/Ks ratios (Figure 2B and Supplementary Table 6) and divergence time for orthologous genes using Ks values (Figure 2C and Supplementary Table 6). The results showed the divergence time of orthologous gene pairs varied from 10.52–102.39 million years between celery and coriander, 19.38–173.48 million years between celery and carrot and 16.40–90.09 million years between coriander and carrot.

### Whole-Genome Duplication (WGD) Played a Leading Role in the Expansion of ARF Gene Family in Apiaceae

**FIGURE 3:**
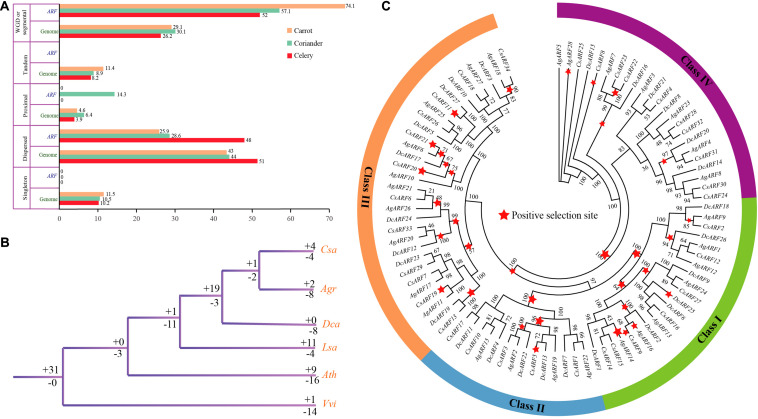
Duplication, loss, and positive selection analysis of *ARF* genes in three Apiaceae species. **(A)** Percentage of different duplication types for *ARF* genes and all other genes. **(B)** Analysis of *ARF* gene duplication and loss. The “ + ” and “–” symbols indicate *ARF* gene duplication and loss, respectively, and the values after these symbols represent gene number. **(C)** Positive selection of *ARF* genes in celery, coriander, and carrot. Red stars represent positive selection branches.

Five gene duplication types were detected, including singleton, dispersed, proximal, tandem, and WGD or segmental duplication (Figure 3A and Supplementary Tables 7, 8). The results indicated that WGD or segmental duplication played a significant role in *ARF* gene family expansion in Apiaceae species. In celery, coriander, and carrot, 52.0, 57.1, and 74.1% of *ARF* genes, respectively, arose by WGD or segmental duplication. No singleton or tandem duplication was detected in *ARF* gene family of these species. Moreover, most *ARF* genes formed collinear blocks within each genome (Supplementary Table 9).

### More Genes Were Lost in Celery and Carrot Than in Coriander After Gene Duplication

In celery, the number of *ARF* genes lost was greater than that duplicated (8 vs. 2), whereas in coriander, the number of *ARF* genes lost and duplicated was equal (Figure 3B and Supplementary Figure 2). Interestingly, in carrot, eight genes were lost, but no gene duplication was detected compared with other species. This phenomenon indicates that more genes were lost after the genome duplication in Apiaceae species. In the common ancestor of coriander and celery, one *ARF* gene duplication and two gene losses were detected. In the common ancestor of coriander, celery, and carrot, 19 genes were duplicated while three genes were lost. This phenomenon indicates that there were more gene duplications in the common ancestor of Apiaceae species compared with the ancestor of lettuce and Apiaceae.

### Most Apiaceae ARF Genes Underwent Positive Selection

Strong positive selection was observed at major nodes of the phylogenetic tree, which may have contributed to the divergence of Apiaceae species. A total of 10, 4, 13, and 5 positive selection branches were detected in class I, II, III, and IV, respectively (Figure 3C). The number of positive selection sites was the highest in class III, indicating that *ARF* genes in class III were under stronger natural selection than those in other classes. Overall, we found that most branches underwent positive selection, which indicates that *ARF* genes played an important role in the evolution of Apiaceae.

### Expression Analysis of ARF Genes

We analyzed the expression patterns of *ARF* genes in three different tissues of celery and coriander. In celery, 16, 9, and 2 *ARF* genes showed higher expression levels in the root, petiole, and leaf, respectively (Supplementary Table 10 and Supplementary Figure 5A). The expression level (FPKM values) of three celery genes (*AgARF9*, *AgARF12*, *AgARF24*) was over 100 in the root. Among all *AgARF* genes, *AgARF9* showed the highest expression level in the petiole. However, *AgARF26* showed no expression in any of the three tissues, while *AgARF1* and *AgARF7* showed no expression in the leaf. Overall, genes in the same phylogenetic group or subgroup showed a similar expression patterns.

In coriander, 9, 22, and 1 *ARF* genes exhibited higher expression in the root, petiole, and leaf, respectively (Supplementary Table 10). Most coriander *ARF* genes were expressed to higher levels in the petiole than in the other two tissues (Supplementary Figure 5B). *CsARF27* showed the highest expression level in the root, while *CsARF12* showed the highest expression level in petiole and leaf. However, *CsARF22* and *CsARF23*, which clustered within the same phylogenetic group, were not expressed in any tissue.

To validate the RNA-seq data, the expression of six *ARF* genes was analyzed by qRT-PCR. The results were consistent with the transcriptome results (Supplementary Figure 6), indicating that our RNA-seq data were reliable.

### ARF Gene Paralogs Exhibit Notably Different Expression Patterns

**FIGURE 4:**
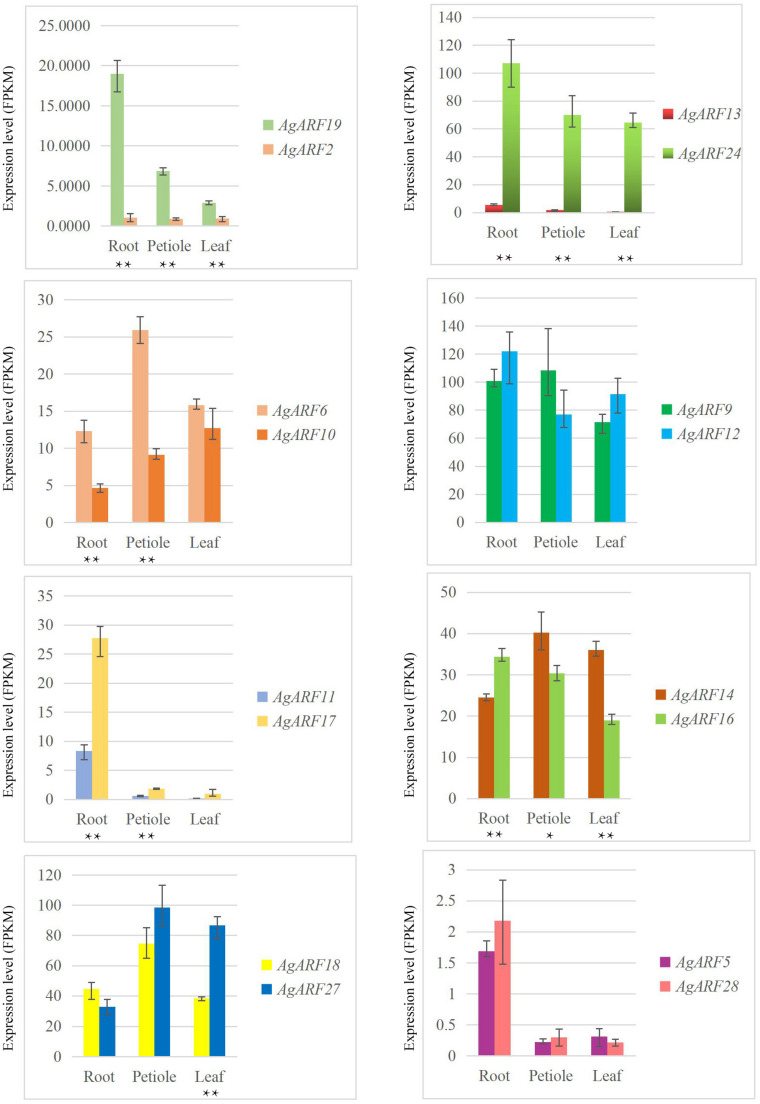
Relative expression level (FPKM) of celery *ARF* paralogous gene pairs in the root, petiole, and leaf. Asterisks indicate significant differences (*P < 0.05, **P < 0.01).

Next, we explored the expression patterns of paralogous genes in celery and coriander (Figure 4 and Supplementary Figure 7). Although most paralogous genes showed similar expression patterns, there were several exceptions. For example, the expression level of *AgARF19* was notably higher than that of *AgARF2* in all three tissues (Figure 4). Similarly, the expression of *AgARF24* was notably higher than that of *AgARF13* in three tissues. In coriander, *CsARF5*, *CsARF7*, *CsARF10*, and *CsARF32* were expressed to higher levels than *CsARF3*, *CsARF13*, *CsARF17*, and *CsARF28*, respectively (Supplementary Figure 7). These results suggest that some paralogous *ARF* genes diverged during the evolution of Apiaceae.

## Discussion

### Functions of ARF Genes in Apiaceae

*ARF* genes exist in most plant species and play key roles in plant growth and development (Li et al., 2016). Most ARF proteins contain three domains: a B3-type DNA-binding domain (DBD) at N-terminus, a variable middle region comprising an activation domain (AD) and a repression domain (RD), and a C-terminal dimerization domain (Tombuloglu, 2019). These domains facilitate interactions with Aux/IAA proteins (Mun et al., 2012; Li et al., 2015). Depending on these domains, ARFs act as transcriptional activators or repressors (Finet et al., 2013).

In Arabidopsis, the function of most *ARFs* has been studied. *ARF1* regulates flower development (Ellis et al., 2005); *ARF2* connects the ethylene and auxin signaling pathways to regulate hypocotyl bending (Okushima et al., 2005); *ARF7* and *ARF19* regulate lateral root formation (Okushima et al., 2007). The characterization of *ARF* gene function in Arabidopsis facilitates the functional analysis of *ARF* genes in Apiaceae. For example, the Apiaceae *ARF* genes in class II clustered together with *AtARF3* and *AtARF4*. Therefore, we speculate that most genes in class II might be related to the floral meristem and reproductive organs. Here, we used bioinformatics to predict the function on a large scale. However, further experimentation is needed to determine the *ARF* gene function in Apiaceae.

Here, we found that some *ARF* gene paralogs exhibit notably different expression patterns, indicating the functions these paralogs diverged during the evolution of Apiaceae. This result is consistent with previous reports on the functional divergence of paralogous genes (Wang et al., 2013a; Soria et al., 2014). In fact, gene duplicates undergo one of four evolutionary fates, including conservation, subfunctionalization, neofunctionalization, and specialization, according to previous reports (Blanc and Wolfe, 2004; Assis and Bachtrog, 2013; Wang et al., 2016). Therefore, this study lays a foundation for further studies on the function of paralogous *ARF* genes in Apiaceae.

### ARFs Interact With Aux/IAA Proteins to Regulate Auxin-Responsive Gene Expression

Both ARF and Aux/IAA proteins act as transcription factors that regulate the expression of auxin-responsive genes (Kumar et al., 2015). *Aux*/*IAA* genes contain several highly conserved domains, and the structure of ARF and Aux/IAA proteins is similar at the C-terminal. Both ARFs and Aux/IAAs harbor the CTD, domain III, and domain IV. Auxin responses rely on ARF–Aux/IAA interactions, which are mediated by CTD (Wang et al., 2013b).

Aux/IAA proteins function as transcriptional repressors, while ARFs function as either activators or repressors for regulating auxin-responsive genes (Liscum and Reed, 2002). The function of ARF proteins is determined by variable middle region (Liscum and Reed, 2002). ARFs are released from Aux/IAAs to repress/activate the expression of auxin-responsive genes (Wu et al., 2017). Here, we identified all *ARF* genes present in three Apiaceae species. This will enable the analysis of interactions between ARF and Aux/IAA proteins in Apiaceae.

### Systematic and Comprehensive Analysis of ARF Gene Family in Apiaceae Species

Although the *ARF* gene family was previously investigated in many plants, there was no report of it in Apiaceae. Whole-genome sequences of celery, coriander, and carrot were released recently (Iorizzo et al., 2016; Song et al., 2020b), which greatly facilitated this study.

To understand the evolution of *ARF* genes, we constructed a phylogenetic tree using ARF amino acid sequences of celery, coriander, carrot, Arabidopsis, lettuce, and grape. Gene structure and conserved motif analyses revealed that genes in the same group or subgroup showed similar features. The number of paralogous *ARF* gene pairs in celery (8) and coriander (12) was notably more than that in carrot (2). Moreover, based on collinearity analysis, we found that WGD had a significant impact on *ARF* gene family expansion in Apiaceae.

In conclusion, we conducted a comprehensive analysis of the *ARF* gene family in three Apiaceae species. Our results provide a strong foundation for comparative and functional analyses of the *ARF* gene family in plants.

## Data Availability Statement

The RNA-seq data were deposited in the Genome Sequence Archive (GSA) of the BIG Data Center (http://bigd.big.ac.cn/gsa) under the accession numbers CRA001996 and CRA001658.

## Author Contributions

XS conceived the project and was responsible for the project initiation. XS and QP supervised and managed the project and research. XS, QP, NL, QY, TW, SF, KG, and TY led the data collection and bioinformatics analyses. XS, QP, NL, XF, ZJ, RZ, and ZW organized, wrote, and revised the manuscript. All authors read and revised the manuscript.

## Conflict of Interest

The authors declare that the research was conducted in the absence of any commercial or financial relationships that could be construed as a potential conflict of interest.

## Footnotes

1 http://cgdb.bio2db.com/

2 http://www.arabidopsis.org/

3 http://bigd.big.ac.cn/gsa
