# Supplementary material for: Genome-Wide Identification and Comparative Analysis of ARF Family Genes in Three Apiaceae Species
Source: Front Genet. 2021 Jan 13;11:590535. doi: 10.3389/fgene.2020.590535 (PMC7838617; doi:10.3389/fgene.2020.590535)
Supplement: Supplementary file 1 [file Data_Sheet_1.PDF]

Supplementary Figures 1-7

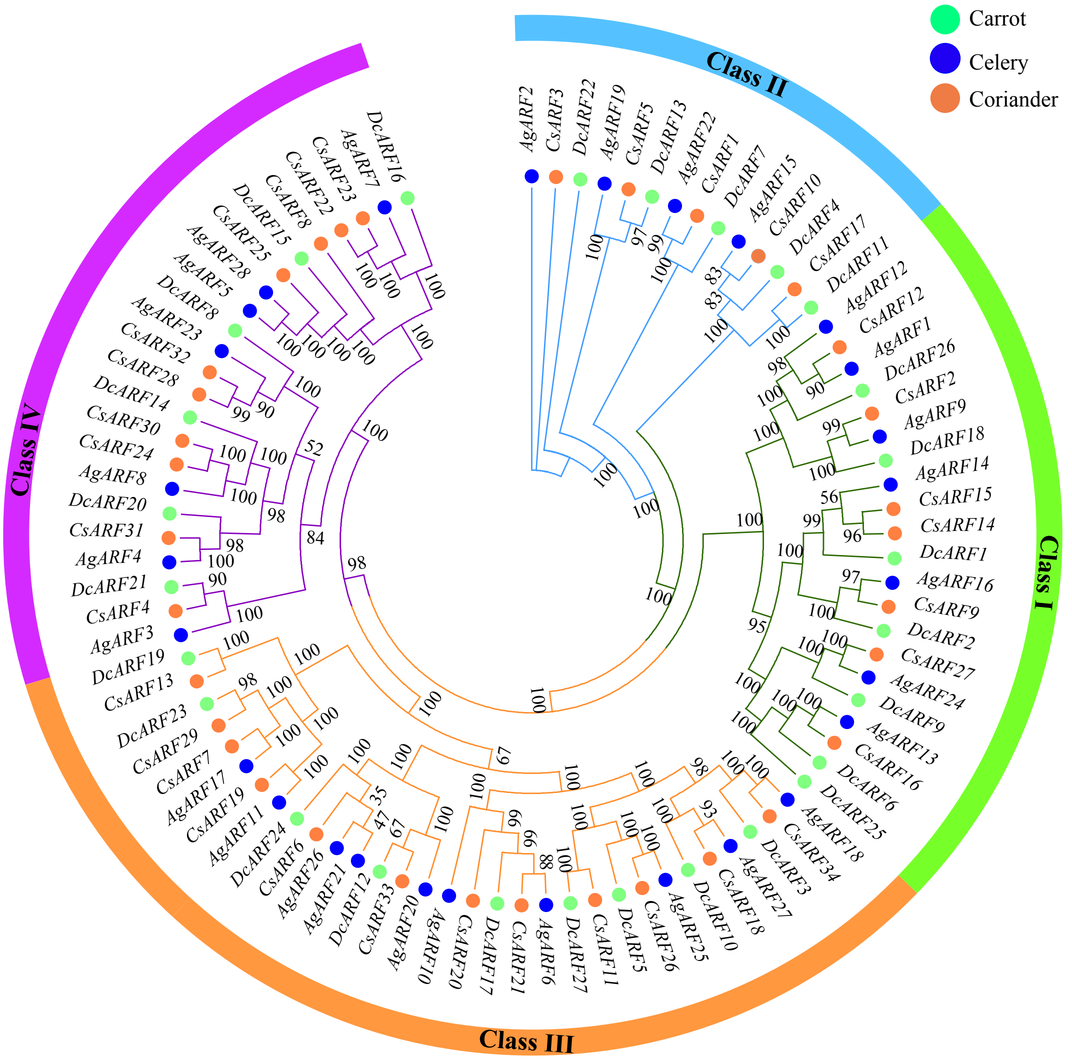

Figure S1. Phylogenetic analysis of the ARF amino acid sequences in celery, coriander and carrot. The phylogenetic tree was generated using the IQ-TREE software with the maximum likelihood (ML) method based on the JTT+F+R8 model and 1,000 bootstrap replications. Classes I to IV were defined based on bootstrap values and phylogenetic topology.

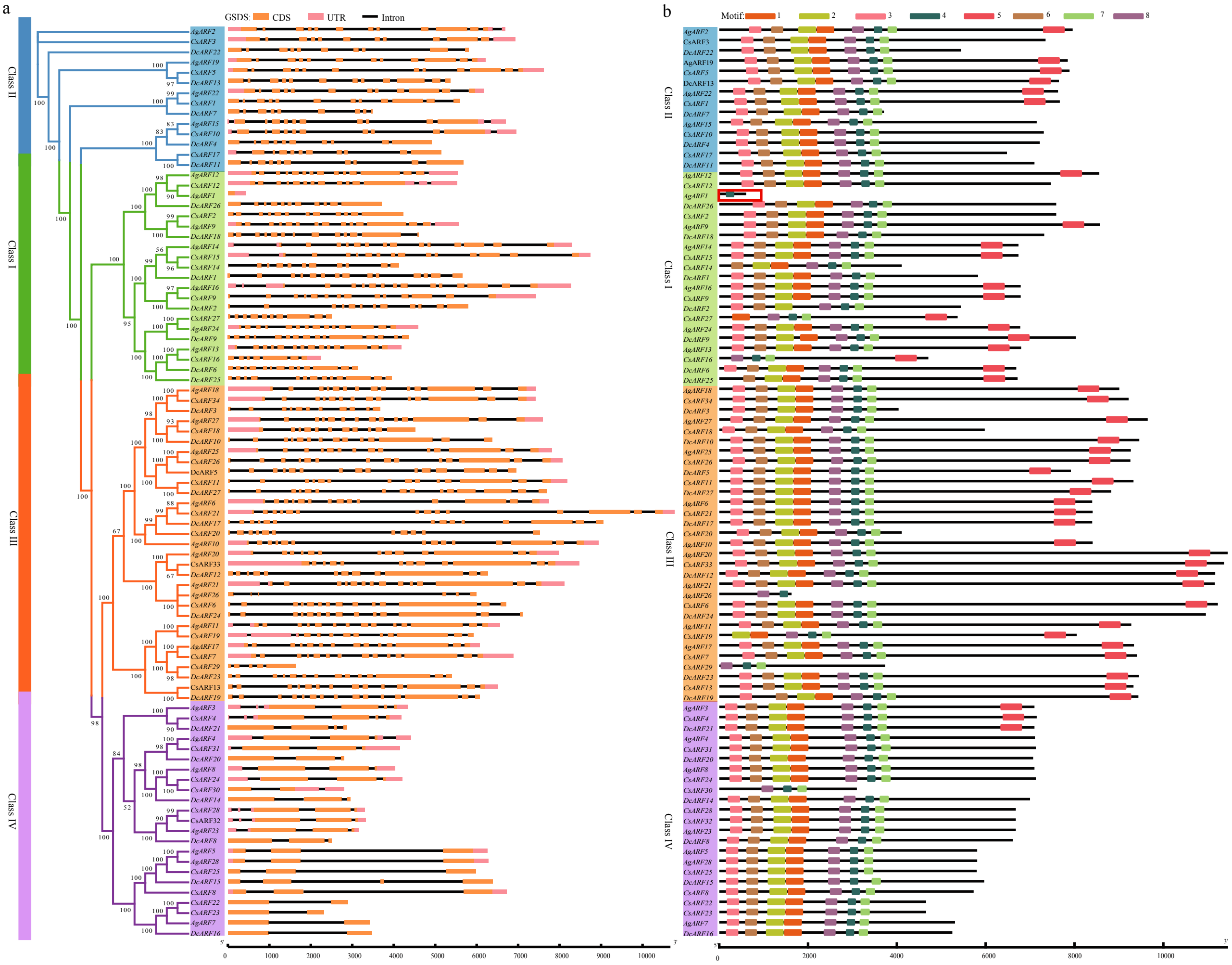

Figure S2. Conserved motif and gene structure analyses of the *ARF* gene family in three Apiaceae species. (a) Conserved motif; (b) gene structure.

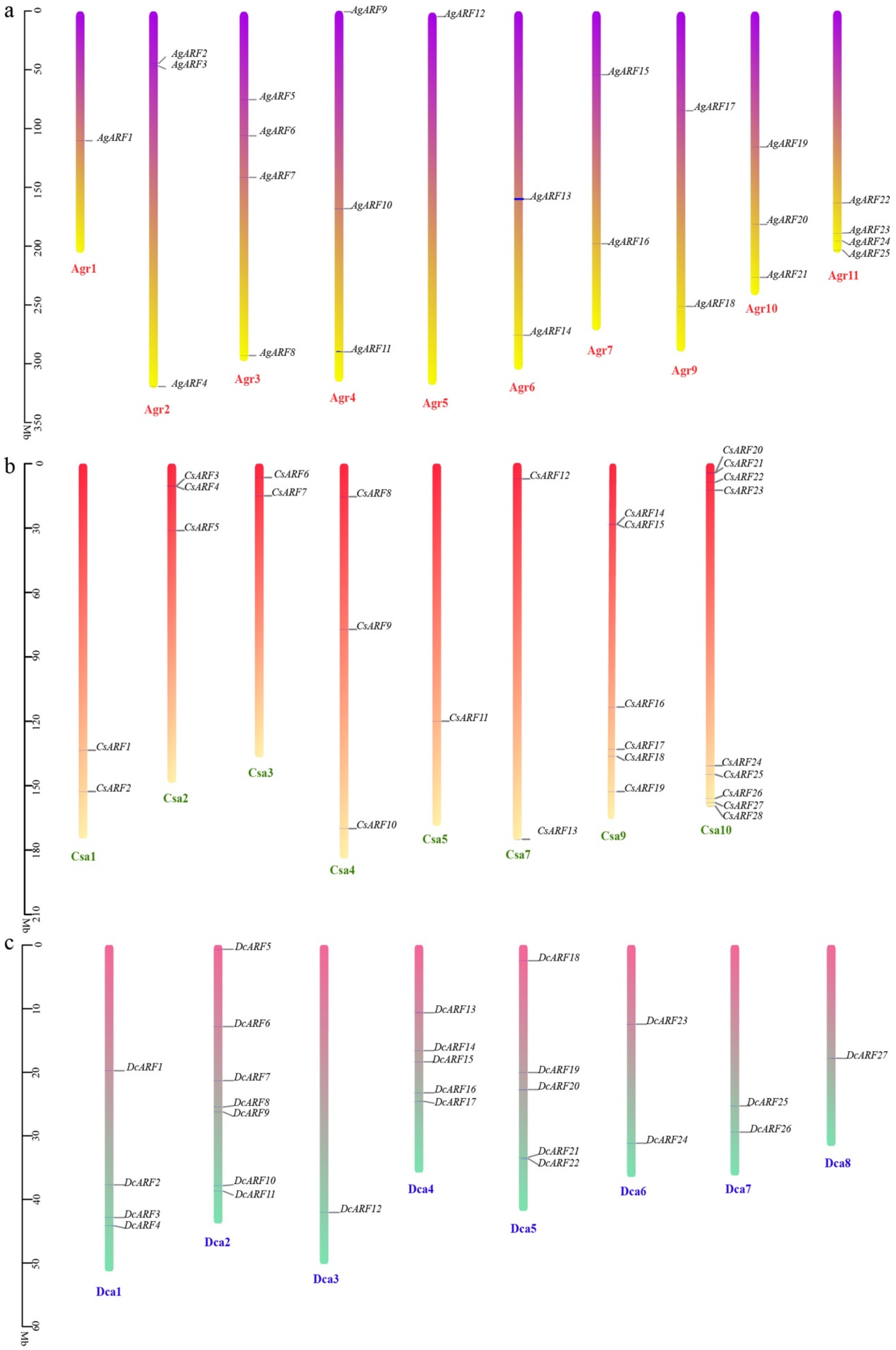

Figure S3. Chromosomal distribution of *ARF* genes in three Apiaceae species. (a–c) Chromosomal distribution of *ARF* genes in celery (a), coriander (b) and carrot (c).

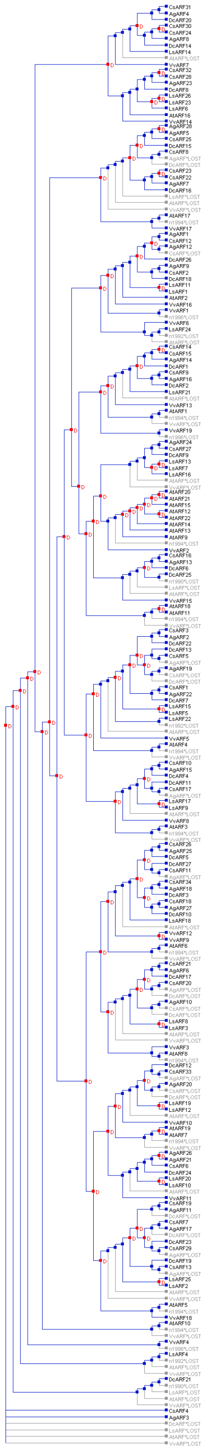

Image generated with Notung 2.9.1, on 2020年1月13日

Figure S4. Reconstructed phylogenetic tree of *ARF* genes in three Apiaceae species (carrot, celery and coriander), lettuce, grape and Arabidopsis. The reconstructed phylogenetic tree was generated using the Notung software through reconciliation between the species and gene trees. Red rectangles or "D" represent duplication. Gene names in gray font represent genes lost during the evolutionary process, and gene names in blue font represent genes that were maintained.

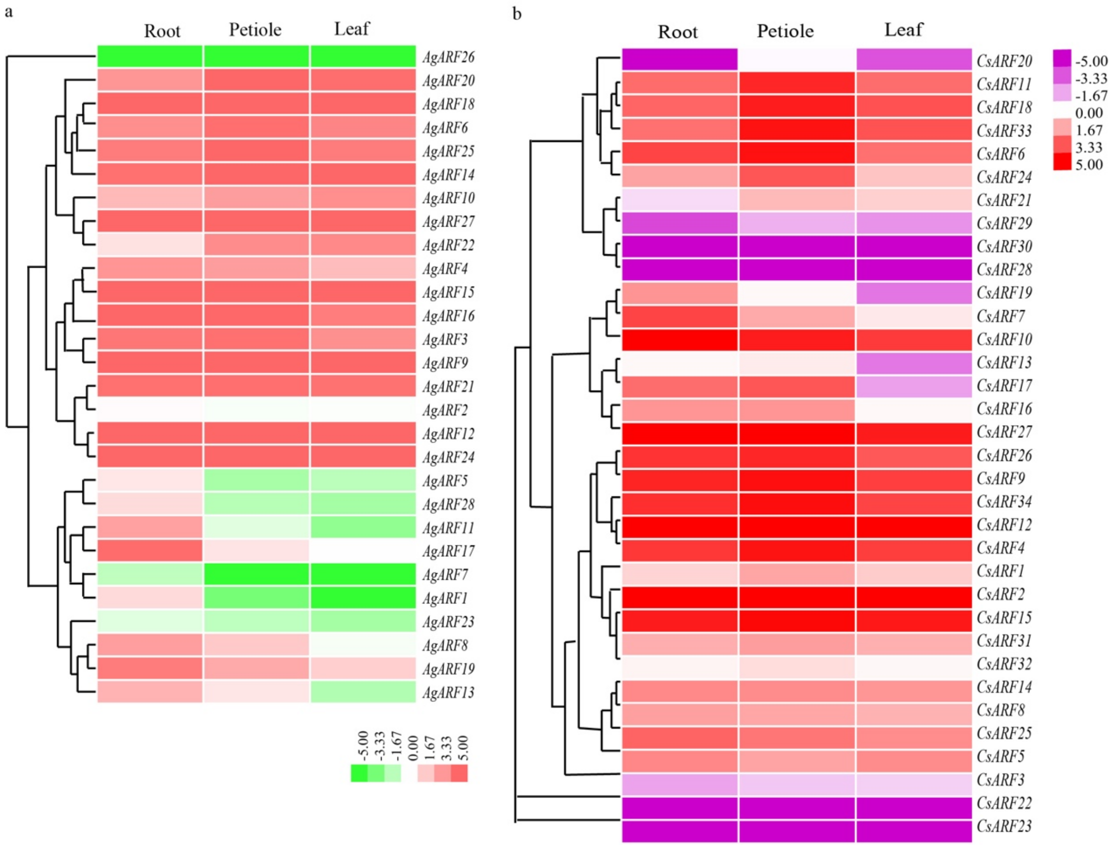

Figure S5. Expression analysis of *ARF* family genes in root, petiole and leaf of coriander and celery. (a, b) Hierarchical clustering analysis of *ARF* genes in celery (a) and coriander (b), according to the RNA-seq data. Gene expression was expressed as FPKM (Fragments Per Kilobase of transcript sequence per Millions base pairs), which were then log2-transformed.

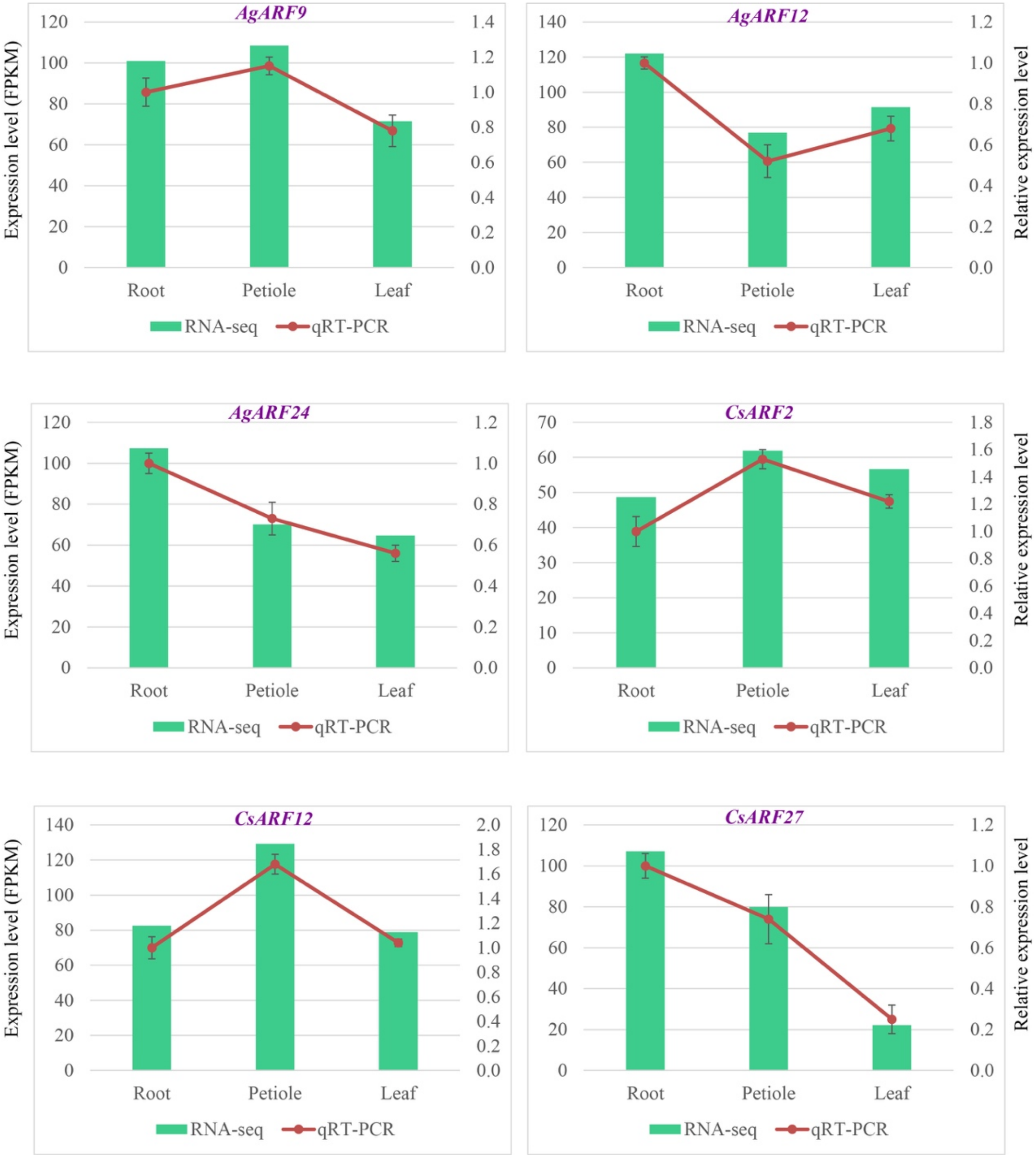

Figure S6. Expression analysis of six *ARF* genes in three different tissues of celery and coriander by quantitative real-time PCR (qRT-PCR). The green histograms indicate gene expression (FPKM) obtained by RNA-seq, and the red line graph represents gene expression obtained by qRT-PCR. Relative gene expression was calculated for petiole and leaf compared with gene expression in the root. Error bars indicate the standard error of three replicates.

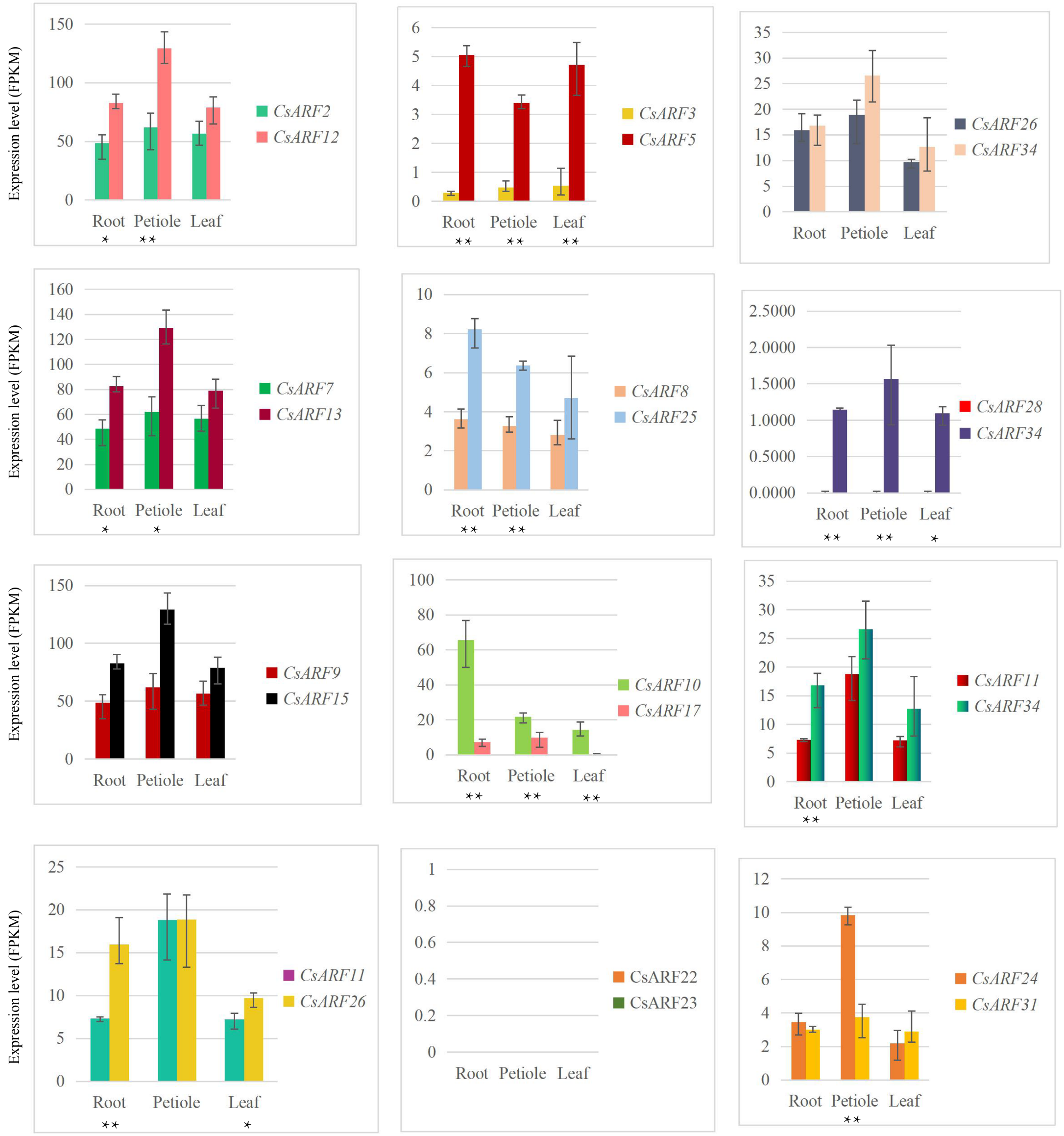

Figure S7. Relative expression level (FPKM) of coriander ARF paralogous gene pairs in root, petiole and leaf tissues. Asterisks indicate significant differences (*$P < 0.05$, **$P < 0.01$).
